# Cancer Stem Cells in Human Gastrointestinal and Hepatic Cancers

**DOI:** 10.1002/mco2.70513

**Published:** 2025-12-03

**Authors:** Jiawen Bu, Mingming Cui, Yu Zhang, Yu Lu, Xudong Zhu, Fu Peng, Hong Zhang

**Affiliations:** ^1^ Department of Colorectal Surgery Shengjing Hospital of China Medical University Shenyang Liaoning China; ^2^ Department of Hepatopancreatobiliary Surgery Cancer Hospital of Dalian University of Technology Cancer Hospital of China Medical University Liaoning Cancer Hospital & Institute Shenyang Liaoning China; ^3^ Liaoning Provincial Key Laboratory of Precision Medicine For Malignant Tumors Liaoning Cancer Hospital & Institute Shenyang Liaoning China; ^4^ Markey Cancer Center University of Kentucky Lexington Kentucky USA; ^5^ Key Laboratory of Drug‐Targeting and Drug Delivery System of the Education Ministry and Sichuan Province Sichuan Engineering Laboratory For Plant‐Sourced Drug and Sichuan Research Center for Drug Precision Industrial Technology West China School of Pharmacy Sichuan University Chengdu China

**Keywords:** cancer stem cells, gastrointestinal cancer, hepatic cancer, stemness, therapeutic strategy

## Abstract

Gastrointestinal cancers, including gastric, hepatic, and colorectal cancers, represent a major public health problem globally. Although the 5‐year survival rate of gastrointestinal tumors has been greatly improved due to the progress of diagnostic and therapeutic strategies, therapeutic resistance and distant metastasis remain the leading causes of cancer‐related death in patients with gastrointestinal tumors. Cancer stem cells (CSCs) are a population of self‐renewal cells with extremely strong carcinogenic potency, contributing to the progression, metastasis, and therapeutic resistance of gastrointestinal tumors. It is generally accepted that gastrointestinal CSCs have the characteristics of self‐renewal, pluripotency, tumorigenicity, metastatic potential, and therapeutic resistance. Hence, understanding the behaviors of gastrointestinal CSCs is important for characterizing the stemness landscapes of gastrointestinal tumors and developing promising strategies for the treatment of gastrointestinal tumors. This review aims to discuss the basic characteristics of gastrointestinal CSCs, existing approaches to define and isolate CSCs, and canonical molecular pathways involved in the regulation of gastrointestinal CSCs. More importantly, potential strategies targeting gastrointestinal CSCs are proposed to overcome the limitations of current therapies.

## Graphical abstract

Gastrointestinal cancer stem cells obtain the ability of unlimited self‐renewal, drug resistance, tumorigenicity as well as tendency of metastasis.

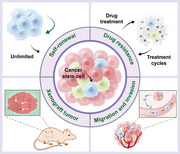

## 1 Introduction

Gastrointestinal cancers, including gastric, hepatic, and colorectal cancers, contribute substantially to cancer‐related death globally. According to the updated cancer statistics, colorectal cancer is predicted to be the third most frequently diagnosed malignancy (8% in male and 7% in female) in North America. Concordantly, colorectal cancer also ranks third in male cancer‐related mortality and the fourth in female cancer‐related mortality [1]. Although the 5‐year survival rate of gastrointestinal and hepatic tumors has been greatly improved due to the rapid progress of diagnostic and therapeutic strategies, therapeutic resistance and distant metastasis remain the leading causes of cancer‐related death in patients with gastrointestinal tumor as well as hepatic tumor. Therefore, recent studies have focused on exploring the molecular mechanisms underlying metastasis and therapeutic resistance of gastrointestinal and hepatic tumors, which may provide more promising therapeutic interventions.

Recent studies have shown that gastrointestinal and hepatic tumors contain a population of cells with extremely strong carcinogenic ability and extremely low differentiation, which are called cancer stem cells (CSCs) due to their characteristics of self‐renewal and multidirectional differentiation [2, 3, 4, 5]. It has been considered to be the initiation factor of cancer occurrence, drug resistance, recurrence, and metastasis, which is generally characterized by self‐renewal, multidirectional differentiation, therapeutic resistance, and maintenance of malignant growth [6, 7, 8]. CSCs often remain after adjuvant treatment with conventional chemotherapy drugs, such as oxaliplatin or capecitabine. These residual tumor stem cells in the cancer niche are similar to seeds, continuously self‐renew and differentiate into recurrent tumor cells, and then mediate the resistance of gastrointestinal and hepatic tumors to adjuvant therapy [3, 9, 10]. Therefore, unraveling the molecular mechanism underlying CSCs and identifying promising therapeutic targets are of great significance for the treatment of gastrointestinal and hepatic tumors.

In this review, we primarily discussed the characteristics of gastrointestinal and hepatic CSCs, including self‐renewal and pluripotency, tumorigenic ability, metastatic potential, and drug resistance. Further, the approaches to identify CSCs of gastrointestinal and hepatic tumors have also been summarized. Next, we discussed the related signaling pathways involved in the regulation of gastrointestinal and hepatic CSCs, such as TGF‐β, Wnt/β‐catenin, JAK/STAT, Notch, and Hedgehog pathways. Moreover, we summarized the therapeutic strategies to eliminate gastrointestinal and hepatic CSCs by targeting surface biomarkers or related signaling pathways.

## 2 Characteristics of Gastrointestinal and Hepatic CSCs

Gastrointestinal and hepatic CSCs are functionally characterized by self‐renewal, pluripotency, tumorigenesis, metastasis, and therapeutic resistance [2, 6, 9]. Here, the unique characteristics of gastrointestinal and hepatic CSCs have been primarily discussed, which may provoke potential therapeutic strategies to target gastrointestinal and hepatic CSCs.

### 2.1 Self‐Renewal and Pluripotency

CSCs are mother cells that could give rise to two daughter cells through asymmetric cell division [11, 12]. Since CSCs were proposed, there were sufficient evidence to confirm the existence in many types of solid tumors, such as colorectal, hepatic, breast, brain, prostate, lung, pancreatic cancers, melanoma, and glioblastoma [6, 13, 14, 15, 16, 17, 18, 19, 20, 21, 22]. Based on CSC hypothesis, researchers believed that not all cells within a tumor are equal, only a small subpopulation of cells, CSCs, possess the unique abilities to self‐renew, differentiate as well as initiate and sustain tumor growth.

Due to self‐renewal and pluripotency, CSCs can divide and form sphere colonies continuously to promote tumor initiation and development [23]. Here lies the critical question. What is the frequency of these highly tumorigenic cells within the overall tumor population? This is where sphere‐limiting dilution assay (LDA) comes in. LDA is a quantitative functional assay designed to determine the frequency of cells within a population that possess a specific capability (in this case, sphere formation in vitro and the ability to initiate tumor growth in vivo). Its core principle involves serially diluting a cell suspension and transplanting these dilutions into a permissive host, such as suspended culture medium or immunodeficient mice. The goal is to find the dilution where only a subset of the transplanted sites develops a sphere over specific diameter or palpable tumor [24]. In practice, to test whether a tumor cell can generate its next generation, cells are dissociated into a single‐cell suspension and then implanted in a suspension culture system with the estimated number of cells from high to low in each culture well or immunodeficient mice (e.g., 10,000 cells, 1000 cells, 100 cells, 10 cells, one cell per injection site, and multiple replicates are used for each dilution) [24, 25, 26]. Those cells that can form a visible spheroid under a microscope or develop palpable tumor in mice are considered to have greater self‐renewal ability [27, 28]. The method of LDA provides direct, quantitative functional data on self‐renewal and pluripotency potential and hence is considered as the “golden standard” for validating CSC properties. However, some disadvantages of this method should not be neglected, such as intensive resources, microenvironment dependence as well as technical challenges.

In all, CSCs are regarded as cells with strong self‐renewal and pluripotency, which could drive neoplastic growth. When evaluating self‐renewal and pluripotency of gastrointestinal and hepatic CSCs, the method of LDA is the primary experimental method used to rigorously quantify the frequency of functional CSCs (obtains the ability to self‐renewal and pluripotency) within a heterogeneous tumor population [29, 30].

### 2.2 Tumorigenic Ability

In 1977, Hamburger and Salmon [31] first discovered that only one in 1000 to one in 5000 cancer cells were able to generate colonies in an in vitro culture system and named these cells as CSCs. The concept was then reintroduced when researchers found that a small population of CD34^+^CD38^–^ leukemic cells could create leukemia in immunosuppressive mice [32]. Over the past decades, the phenomenon that a small population of cells can give rise to tumor formation has been described in various solid tumors such as pancreatic carcinoma [33], hepatocellular carcinoma [34], retinoblastoma [35], breast cancer [36, 37], bladder cancer [38], prostate cancer [39], and colorectal cancer [7]. Since then, detecting the tumorigenic ability of specific cells has been the golden standard for evaluating the stemness of cancer cells. Normally, following the principle of LDA, when implanting cells into immunosuppressive mice, up to 10^4^–10^7^ cells are sufficient for tumor formation depending on different cancer cell types [18, 40]. However, when it comes to CSCs, much fewer cells are required for tumor formation. Du et al. [41] reported that as few as 100 CD44^+^ primary human colorectal cancer cells could initiate xenograft tumor in vivo, whereas the number of CD44^−^ cells with a similar ability exceeded 10,000. Thus, tumorigenic ability can be regarded as the golden standard for detecting the stemness of cancer cells.

### 2.3 Metastatic Potential

Higher expression of stem cell markers has been found in primary tumors or metastatic sites of gastrointestinal tumors [42, 43]. CSCs of gastrointestinal tumors display significantly enhanced migration and invasion ability compared with non‐stem cells [44, 45]. In an established orthotopic mouse model of colorectal cancer, organoids with an enhanced stemness phenotype (Lgr5^+^) disseminated to liver as early as 3 weeks after injection. In contrast, the orthotopically implanted organoids with impaired stemness failed to disseminate into distant organs even after 6 weeks of implantation [46]. Li et al. [47] deciphered the landscape of heterogenous cancer stem‐like cells in colorectal cancer and their organ‐specific metastasis through single‐cell transcriptomic analysis. In 2020, Ganesh et al. [48] discovered that L1CAM^+^ cells partially overlaps with Lgr5^+^ CSCs and L1CAM mediates the perivascular spreading of disseminated metastatic cells in distant organs via intimate interactions between the basal lamina of the endothelium and metastatic cells, suggesting the metastatic potential of colorectal CSCs. Dclk1 also marks the stemness of colorectal cancer, researchers found that when depleting Dclk1^+^ stem cells in colorectal cancer, liver metastases were significantly inhibited [49]. Single‐cell transcriptomics of colorectal cancer liver metastases found a group of stem‐like cells, which expressed genes associated with stem cell‐like characteristics and metastatic potential [50]. At the same time, CSCs are also recognized as pivotal players in the progression and metastasis in hepatic cancer. Growing evidences indicate that epithelial–mesenchymal transition (EMT) in gastrointestinal and hepatic cancer cells is always accompanied by the acquisition of stemness and some EMT inhibiting drugs were found to inhibit stem cell properties as well [51, 52, 53, 54, 55, 56, 57]. For example, TGF‐β1 and HIF‐1α were reportedly induced the EMT to promote hepatic cancer cells transforming into the CSC phenotype [58, 59]. Choi et al. [60] reported that stem cell markers and EMT markers were closely correlated with each other in colorectal adenocarcinoma. The expression level of EMT markers (E‐cadherin, β‐catenin, Snail, Vimentin) was correlated with the expression level of stem cell surface marker CD133 and facilitated cancer cell invasion as well as metastasis. Environmental inflammatory factors such as TGF‐β1, chemokines, interleukin (IL)‐17, lipopolysaccharide, IL‐6, and HGF exist and play an important role in the regulation of non‐CSC acquired stemness or CSC maintenance stemness in the hepatic cancer cell tumor microenvironment (TME) [61, 62, 63, 64, 65].

With respect to the reasons for higher metastatic potential of gastrointestinal and hepatic CSCs, researchers conclude reasons as follows. CSCs could become metastasis‐initiating cells by several cellular processes, including EMT, CSC niche interactions, and extracellular matrix (ECM) remodeling, then intravasate into the blood vessels, retaining their original stem cell phenotype, and CSCs can generate heterogeneous tumor cells, enabling adaptation to new microenvironments, survive in the blood or other harsh environments through dormancy, sustained quiescence, and metabolic reprogramming [66].

### 2.4 Drug Resistance Phenotype

Chemotherapeutic regimens have remarkably improved the survival rate of gastrointestinal and hepatic tumors [67]. However, the phenomenon of chemotherapeutic resistance remains a huge barrier to the systemic treatment of gastrointestinal tumors [68]. Gastrointestinal CSCs possess the ability to evade the killing effect of DNA‐damaging chemotherapy [69]. The DNA‐damaging repair functions of gastrointestinal CSCs are also more powerful than normal cancer cells due to the expression of DNA‐damaging repair proteins, such as poly (ADP‐ribose) polymerase (PARP), hence sustaining the survival of tumor cells under the pressure of DNA‐damaging drugs [70]. Patients with colorectal cancer who gain limited benefit from chemotherapy usually exhibit a higher expression of stem cell markers [69]. Todaro et al. [71] found that both oxaliplatin and 5‐fluorouracil are less effective in CD133^+^ colorectal cancer cells compared with CD133^−^ cells. The increased fraction of CD133^+^ cells in residual tumors after chemotherapy implies that most non‐stem cells can be eliminated by chemotherapy while CSCs survive [71]. This phenomenon has been explained by the theory that current cytotoxic agents are designed to kill actively proliferating cells. Compared with non‐stem cells, CSCs may escape chemotherapy as they are relatively quiescent [72]. Besides, colorectal CSCs can also pump out cytotoxic reagents by upregulating the expression of the ATP‐binding cassette (ABC) family proteins [73]. A variety of signaling pathways that regulate self‐renewal and cell survival (such as WNT, Notch, TGF‐β, and p53) are dysregulated in gastrointestinal CSCs, thus contributing to the drug resistance to chemotherapy [74]. The dysregulation of metabolic processes (such as overexpression of ALDH and enhanced ROS level) in CSCs may also play a role in therapeutic resistance [75]. Emerging evidence indicates that gastrointestinal CSCs also play important roles in therapeutic resistance to immune checkpoint blockades. Zheng et al. [76] characterized the stem cell landscape and proposed a stemness‐related prognostic gene signature to aid immunotherapy in colorectal cancers. Besides, gastrointestinal CSCs participate in the regulation of the immune microenvironment by various signaling molecules [77]. Therefore, targeting gastrointestinal CSCs may achieve highly effective tumor immunotherapy. Meanwhile, CSCs could also change their own molecular expression and reprogram the immune response to maintain the tumorigenic process and induce the drug resistance phenotype in hepatic cancer [78]. EpCAM^+^ liver CSCs were reportedly upregulating CEACAM1 expression to resist natural killer (NK) cell‐mediated cytotoxicity [79]. In fact, researchers have already discovered side population (SP) cells and several surface markers, such as EpCAM, CD133, CD44, CD13, CD90, CD24, CD47, OV6, K19, c‐kit, ABCG2, and ALDH, which are identified influencing drug efflux‐related gene expression, activation of growth signaling, and stem cell‐related and antiapoptosis pathways to affect the resistance to radiotherapy or chemotherapy [80, 81, 82, 83, 84, 85, 86].

In gastrointestinal and hepatic cancers, CSCs do not represent a static or fixed cellular subpopulation. Increasing in vivo and in vitro evidence indicate that CSC states are highly dynamic and can reversibly interconvert among tumor cells. Fumagalli et al. [87] demonstrated in animal models that circulating and disseminated colorectal cancer cells predominantly exhibit an Lgr5^−^ phenotype, while Lgr5⁺ CSCs reappear at metastatic sites, suggesting that non‐CSCs can reacquire stem‐like features under appropriate stimuli and initiate metastatic outgrowth. Moreover, Wang et al. [8] measure the telomere length and transcriptome in single cells to systematically characterize CSC states in primary colorectal cancer, revealing that rare CSCs exist in a dormant state and display bidirectional plasticity toward cancer epithelial cells, which may serve as presumptive tumor‐initiating cells. In the study of Centonze et al., [88] they reveal a novel mechanism through which metastatic colorectal cancer (mCRC) develops resistance to KRAS–G12D inhibitors: tumor cells rapidly undergo plastic conversion from an EMP1⁺ non‐stem cell state to an Lgr5⁺ stem‐like state to evade drug treatment. Mechanistically, KRAS–G12D signaling itself suppressed the Wnt‐driven Lgr5⁺ stem cell program. When KRAS is strongly inhibited by drugs like RMC‐9945, this “brake” is released. The previously suppressed Lgr5⁺ program is activated, causing cells to rapidly convert from the EMP1⁺ non‐stem cell state (associated with metastasis and poor prognosis) to the Lgr5⁺ stem‐like state with high survival and proliferative capacity, thus resulting in drug resistance [88]. Apart from the transition of non‐CSCs to CSCs, CSCs also have cell state transition. For instance, some CSCs are slow cycling and drug resistant (revival CSCs), whereas another part of the CSCs is fast cycling and drug sensitive (proliferative CSCs). CSCs exist in a dynamic state and can interconvert between revival CSCs and proliferative CSCs under niche signals such as TGF‐β, YAP, Wnt, MAPK, and PI3K signal pathway [89, 90].

Traditional therapies often fail because cells exist in a dynamic equilibrium, transitioning between distinct functional states, these shifts allow cancer cells to evade treatment, leading to tumor persistence and relapse. Based on the understanding of the plasticity of CSCs, potential therapeutic strategies were also raised by researchers. To target this dynamic system, therapeutic approaches shall prevent non‐CSCs from acquiring stem‐like properties and restoring the cancer stem pool. Research in colorectal cancer models reveals that chemotherapy‐resistant, dormant Lgr5⁺p27⁺ CSCs reignite tumor growth through the COL17A1–FAK–YAP pathway. To prevent this, pharmacological inhibition of FAK or YAP‐via small‐molecule inhibitors or knockdown—successfully suppressed the exit from dormancy and delayed regrowth in preclinical models. Consequently, combining standard chemotherapy with drugs that target these reactivation signals presents a viable strategy to counteract CSC plasticity and suppress recurrence [91]. Building on the theme of cellular plasticity, Coppo et al. [92] characterized a slow‐cycling, drug‐tolerant subpopulation (S‐cells) within patient‐derived organoids (PDOs) that persisted after treatment with 5‐FU and the MEK inhibitor PD0325901. Following drug withdrawal, these cells regained proliferative capacity through a Notch/MSI1‐dependent mechanism. Critically, using the γ‐secretase inhibitor DAPT to pharmacologically block Notch signaling prevented this regenerative transition and suppressed tumor regrowth. These results advocate for a shift in therapeutic strategy from solely targeting proliferation to inhibiting the plasticity‐driven transitions that fuel relapse, nominating the Notch/MSI1 axis as a promising therapeutic target [92]. However, unlike previous examples, the team of Centonze et al. [88] designed an “induce‐and‐eliminate” combination therapy strategy. First, they used the KRAS inhibitor RMC‐9945 to force tumor cells into the Lgr5⁺ state. Subsequently, genetic methods were used to specifically eliminate Lgr5⁺ cells. In mouse models, this strategy significantly reduced metastatic burden and prolonged survival, providing strong proof‐of‐concept for future development of combination therapies [88].

**FIGURE 1:**
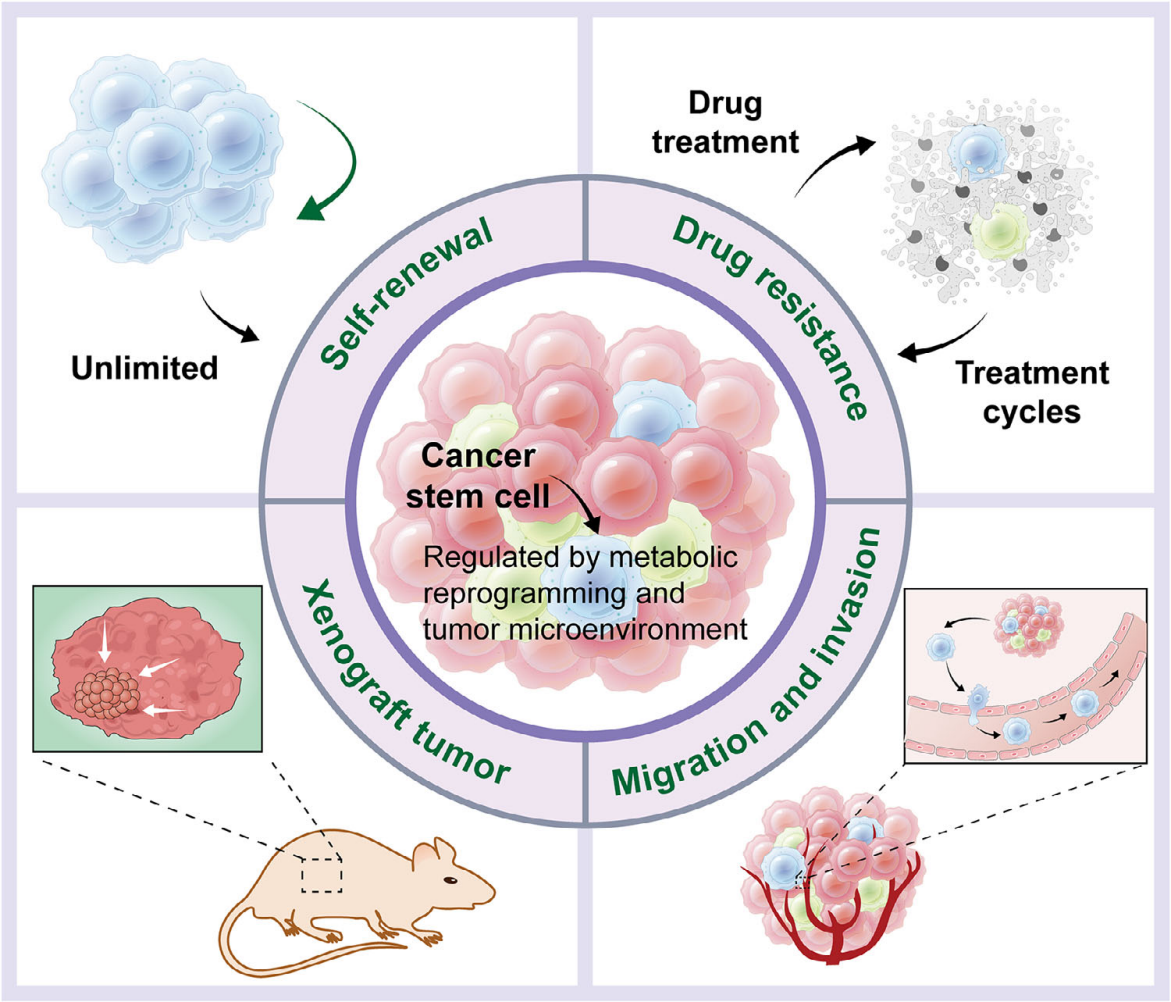
Ideograph for unique characteristics of gastrointestinal CSCs. Gastrointestinal CSCs obtain the ability of unlimited self‐renewal, drug resistance, tumorigenicity, tendency of metastasis as well as changes in metabolic feature.

In short, gastrointestinal and hepatic CSCs possess the unique drug‐resistant ability. They can increase the survival rate through multiple mechanisms such as overexpression of DNA repair proteins, remaining relatively quiescent allows escape from chemotherapy targeting proliferating cells, upregulation of ABC transporters actively effluxes cytotoxic drugs, dysregulation of stemness pathways and metabolic adaptations, which sustain self‐renewal and survival. After chemotherapy, residual CSCs can cause tumor relapse and metastasis through phenotypic plasticity or by inducing the reprogramming of non‐CSCs. Furthermore, gastrointestinal and hepatic CSCs participate in regulating the immune microenvironment and mediate resistance to immune checkpoint inhibitors. Targeting gastrointestinal and hepatic CSCs and their associated pathways hold promise for overcoming current therapeutic limitations. The ideograph for characteristics of gastrointestinal CSCs was also summarized in **Figure** **1**.

### 2.5 Roles of TME in Regulating Properties of CSCs

As the cognition for gastrointestinal and hepatic CSCs gradually deepens, researchers have realized that the in vivo existence as well as the maintenance of stem cell properties were not only relied on intrinsic signaling pathways of CSCs, but also on the TME. In this section, we generally elaborated the roles of TME in regulating stemness maintenance, cell plasticity, metastatic potential, and drug resistance ability of CSCs.

When it comes to the roles of TME in CSCs, one major component that constitutes the formation of TME may never be neglected, that is cancer‐associated fibroblasts (CAFs). In colorectal cancer, CAFs increased the stemness and the migration ability of cancer cells in liver or lung metastasis by upregulating CD44 expression through HGF/MET/AKT signal pathway [93]. Chen et al. [94] revealed that CAF‐secreted SDF‐1 significantly enhanced colorectal cancer cell migration, invasion as well as stemness through paracrine signaling. In gastric cancer, downregulation of CAF‐derived secreted protein acidic and rich in cysteine can lead to dedifferentiation of gastric cancer cells into CD44^+^/CD24^−^ CSCs via AKT/mTOR signaling pathway [95]. In the research of Yawen et al. [65], CAF‐derived HGF and IL6 enhanced the stemness properties of CD24^+^ hepatic CSCs via STAT3 signaling pathway. Colella et al. [96] found that colorectal CSCs can secret small extra cellular vesicles under the control of CD147, which may result in the activation of aerobic glycolysis and increased lactate release of CAFs. These finally caused increased cytokines production and VEGF release as well as increased cancer invasiveness in vitro and tumor growth in vivo [96]. Apart from regulating the stemness and cell plasticity of CSCs, CAFs may also affect drug resistance phenotype of gastrointestinal and hepatic CSCs. CAF‐secreted proteins such as SPP1, periostin, and COL8A1 can promote tumor cell EMT as well as cancer stemness and lead to resistance to chemotherapy or tyrosine kinase inhibitors treatment [97, 98]. Furthermore, CAFs extensively remodel the ECM, creating a physical barrier that impedes drug penetration and immune cell infiltration, thereby fostering a protective niche for CSCs [99]. Tumor‐associated macrophages (TAMs) are another vital component of the TME. Gastrointestinal and hepatic CSCs actively recruit macrophages and shape their polarization toward tumor‐promoting phenotypes through chemokines and cytokines. In return, TAMs support and expand CSC properties through multiple mechanisms, including the secretion of IL‐6, TGF‐β, MFG‐E8 and extracellular vesicles, direct cell–cell interactions, and remodeling of ECM, ultimately reinforcing cancer stemness and tumor malignancy [58, 64, 100].

### 2.6 Roles of Metabolic Reprogramming in Regulating Properties of CSCs

Metabolic reprogramming is also a crucial hallmark of gastrointestinal and hepatic CSCs. CSCs obtain the unique metabolic feature compared with non‐CSCs in order to maintain self‐renewal, drug‐resistant, and invasive ability. For instance, aldehyde dehydrogenase (ALDH) is an enzyme responsible for the oxidation of intracellular aldehydes as well as a marker for the isolation of CSCs. Mechanistically, ALDH1 is proposed to have the ability to maintain CSC characteristics through retinoic acid metabolism [101]. Moreover, high expression of ALDH helps CSCs metabolize pharmaceuticals and other pollutants thus increasing the ability of drug resistance. In colorectal cancer, the ALDH proteins, especially ALDH1A1and ALDH1A3, contribute to the chemoresistance as well as increased CSC markers such as CD133, CD166, CD24, CXCR4, CD26, CD271, and CD274 [102, 103]. Traditional theories believed that the malignancy of tumor cells were correlated with increased Warburg effect, which is also known as glycolysis [104]. However, recent evidence indicates that stem‐like populations can switch between glycolysis and oxidative phosphorylation (OXPHOS) depending on the oxygen and nutrient supply, enabling them to overcome therapy‐induced stress and maintain tumorigenic potential in gastrointestinal and hepatic CSCs [105]. For instance, in colorectal cancer, some cancer stem populations display a hybrid metabolic profile. Upregulation of glycolysis is observed when glucose is abundant, while as glucose decreases, β‐oxidation of fatty acids or OXPHOS become the mainstream of metabolic pathways [105]. Notably, CSCs that rely on OXPHOS can maintain robust ATP production even when glycolytic intermediates or glucose are scarce, allowing them to survive chemotherapy that targets rapidly proliferating, highly glycolytic non‐CSCs [106]. Apart from the changes in glucose intake, some CSC subpopulations rely on glutamine as an alternative nutrient source. In pancreatic ductal adenocarcinoma (PDAC), CSCs exhibit a pronounced dependency on glutamine, which helps fulfilling intermediates in TCA cycle, fueling CSC proliferation as well as protecting CSCs from oxidative stress [107]. Besides, lipid metabolism takes part in the maintaining of CSCs as well. Adipocytes and CAFs in TME can secret free fatty acids, which could be utilized by CSCs via transporters fatty acid such as CD36 and FATP2 to sustain fatty acid oxidation (FAO) [108]. This metabolic feature not only provides energy for CSCs, but also confers resistance to chemotherapy‐induced stress. Consequently, targeting lipid metabolism using FAO inhibitors can impair the survival of CSCs as well as sensitizing them to chemotherapy [108].

## 3 Available Approaches to Identify Gastrointestinal and Hepatic CSCs

The identification of CSCs provides a powerful tool for a better understanding of tumor initiation and development. Researchers can define gastrointestinal and hepatic CSCs based on their specifically expressed surface markers, unique population characteristics in flow‐cytometry, self‐renewal ability when cultured in a suspension medium, or even the high expression of certain metabolic enzymes [109]. CSCs can be identified and separated by functional assays, such as ALDH activity assay and floating spheres, as well as CSC‐specific cell surface marker expression.

### 3.1 Identifying CSCs via Surface Markers

CSCs can be isolated and identified by CSC‐specific cell surface marker expression [109, 110]. Due to their specific expression on the cell surface, it is relatively easy to label and isolate CSCs with coordinating antibodies. To date, a series of surface markers are implicated in gastrointestinal and hepatic CSCs, such as CD133, CD44, Epcam, Lgr5, Dclk1, CD166, CD49f, CD24, SOAT1, EphA2, and EphB2 [41, 46, 109, 110, 111, 112]. CD133 is a transmembrane glycoprotein that mainly localizes to membrane protrusions and can be used as an essential marker to identify CSCs. In 2007, O'Brien et al. [18] identified a group of colon cancer‐initiating cells with high expression of CD133 by using renal capsule transplantation in immunodeficient mice. Moreover, cancer‐initiating cells in the CD133^+^ population maintain themselves upon serial transplantation, making CD133 a canonical marker of CSCs [18]. CD133^+^ hepatocellular carcinoma cell population exhibited therapeutic resistance to chemotherapeutic agents by activating Akt signaling pathway as well as Wnt/β‐catenin pathway [113, 114]. As a transmembrane glycoprotein and a ligand of hyaluronic acid, CD44 is considered to be a robust marker of CSCs in multiple solid tumors such as bladder cancer, pancreatic cancer, prostate cancer, breast cancer, and gastrointestinal tumors [41, 115, 116]. Dalerba et al. [6] reported that Epacm^high^/CD44^+^ cells in primary colorectal cancer have self‐renewal ability and can be regarded as CSCs. Moreover, CD44 could maintain cancer stemness and CD44 knockdown can remarkably impair sphere formation and tumorigenicity in the xenograft model of colorectal CSCs [41]. Epcam is a canonical marker for epithelial cells and can be expressed in epithelial carcinoma. Epcam alone is not sufficient to identify gastrointestinal CSCs, which is required to coordinate with CD44 [117]. Epcam^+^ cells are also highly expressed in aggressive hepatocellular carcinoma subtypes, correlating with poor prognosis. These cells also display stem‐like features, such as differentiation plasticity and miR‐155 overexpression, which sustains their survival both in vitro and in vivo [118]. Leucine‐rich repeat‐containing G‐protein‐coupled receptor (LGR5) is a seven‐transmembrane protein and a marker of colorectal CSCs, which can potentiate canonical Wnt/β‐catenin signaling [119]. Barker et al. [120] revealed that Lgr5^+^ cells marked stem‐like cells in the normal intestinal and clonal crypt. Using the established Lgr5‐deficient mouse model, they also demonstrated that Lgr5^+^ crypt stem cells are the cells of origin of intestinal cancer. Dclk1 was first described as a brain‐specific protein in developing rodent brain and functionally associated with microtubules and overexpression of Dclk1 stimulated microtubule elongation [121, 122]. During the past decades, researchers found that Dclk1 might also define CSCs in APC^min^ adenomas. In 2013, Chiba and coworkers found that in APC^min^ mouse model of colorectal cancer, Dclk1^+^ tumor cells fulfilled all bona fide criteria of tumor stem cells continuously fueling adenoma growth and also expressed the known intestinal stem cell marker Lgr5, targeted depletion of Dclk1^+^ cells led to a significant regression of established adenoma [123]. Those results suggest the functional roles of Dclk1 in tumor initiation. CD166 belongs to the subfamily of immunoglobulin receptors, which can bind to T‐cell differentiation antigen CD6 and widely participate in tumor metastasis [124]. CD166 often coordinates with CD44 or CD133 to identify colorectal CSCs [110]. HARAGUCHI et al. [125] found that the coexpression of CD166 and Epcam indicated higher tumor stage, more invasive biological behaviors as well as increasing cancer stemness. CD49f is a member of the integrin family, which is also known as ITGA6. CD49f acts as a functional marker for maintaining the stemness of colorectal cancer cells, and CD49f^+^/CD44^+^ cells display strong capabilities of self‐renewal and multidifferentiation [125]. In hepatocellular carcinoma, CD49f also plays a prominent role in maintaining tumor‐initiating cells; CD49f‐high tumor‐initiating cells specifically recruit tumor‐promoting neutrophils via the CXCL2–CXCR2 axis and create an immunosuppressive milieu in the TME, thus sustaining tumor growth [126]. CD24 is a cell adhesion molecule and is first identified as a stem cell marker in pancreatic and breast cancer [15, 33]. In colorectal cancer, CD133^+^/CD24^+^ cells exhibit great clonogenic potential and multilineage differentiation, the expression of CD133 and CD24 also correlates with the expression of EGFR, KRAS, Ki67 as well as stemness‐related signaling pathways such as Wnt/β‐catenin and Hedgehog signaling pathways [127, 128]. With the help of novel proteomics, some novel therapeutic targets of hepatocellular carcinoma CSCs were indentified. SOAT1 is also newly identified as a cholesterol metabolism regulator in aggressive hepatocellular carcinoma subtypes, which maintains membrane cholesterol levels to support CSC proliferation and migration [129]. Tyrosine kinase family members also play important roles in tumor stemness and signaling transduction in various solid tumors [130, 131]. In colorectal cancer, EphA2 and EphB2, belonging to the tyrosine kinase receptor family, have been identified as novel markers for colorectal CSCs, whose expression is strongly correlated with the expression of CD44 and Lgr5, as well as the stemness of colorectal cancer cells [111, 112]. The summarization of surface markers of gastrointestinal and hepatic CSCs is also illustrated in Table 1 [132].

**TABLE 1:** Surface markers of gastrointestinal CSCs.

Surface markers	Related signaling pathways	References
CD44	Activation of AKT pathway	[41, 93, 94]
CD133	Activation of AKT pathway	[18, 91]
LGR5	Activation of Wnt/β‐catenin pathway	[97]
Dclk1	Activation of Notch, Wnt/β‐catenin pathway	[110]
CD166	Activation of EGFR/ERK pathway	[102]
CD49f	Activation of CXCL2–CXCR2 axis	[104]
CD24	Activation of Hedgehog pathway	[15, 33]
EphA2/B2	Activation of JAK/STAT and AKT pathways	[89, 90]

### 3.2 Identifying CSCs via SP Assay

Cancer SP represents a subset of CSCs that play an important role in therapeutic resistance due to their increased expression of the ABC family proteins involved in the export of cytotoxic small molecules. Thus, the SP assay has been used as a critical technique for the identification and isolation of CSCs. Based on the Hoechst dye staining assay, colorectal CSCs have a significantly weaker intake of Hoechst, and this fraction of cells is manifested as SP [133]. Haraguchi et al. [83] reported the SP method of isolating CSCs from gastrointestinal cancer cell lines since it has been wildly used. Feng et al. [134] reported that combination of verapamil with Hoechst dye could reduce the ratio of SP cells and sort out cells with stronger stemness. Those “refined” SP cells are able to generate more tumor spheres and CD133 positive. Moreover, they exhibited marked multidrug resistance and enhanced cell survival rates compared with non‐SP cells [134]. However, there are still some challenges in the practice of using the SP method, including the parameters of the SP protocol, the concentration of Hoechst, and the specificity to distinguish CSCs from differentiated cells [135]. Hence, when applying the SP method, the parameters should be critically set for analyzing different cell lines or primary tumor tissues. Additionally, given that the SP method does not encompass the entirety of the stem cell population, it is essential to develop additional methods to enrich this stem cell source for comprehensive characterization.

### 3.3 Identifying CSCs via Enzyme Activity Assay

ALDH1 is an enzyme responsible for the oxidation of intracellular aldehydes, which is essential for the early differentiation of stem cells. The ALDH family in human genome contains 19 ALDH genes, ALDH1A1 isoform is commonly considered to be responsible for increasing ALDH activity in caner stem cells, although recent studies have shown that other isoforms contribute to increased activity, in particular the ALDH1A3 isoform [136]. As for gastrointestinal cancers, researchers have found that both ALDH1A1 and ALDH1A3 contributed to the fraction of ALDH^+^ cells [137, 138]. The measurement of ALDH1 activity was mainly relied on ALDEFLUOR assay, which was developed by STEMCELL Technologies, which brings a promising and universal approach to identify and isolate CSCs in the context of different cancers [36, 137, 139]. Compared with CD44^+^ stem cells or CD133^+^ stem cells, the proportion of ALDH^+^ cells in colorectal cancer is quite low, while cells with stronger tumor‐initiating properties can be selected through isolation by combining these markers together [137]. As a single marker, the enzymatic activity of ALDH1 can be changed by chemotherapeutic agents. Therefore, enzyme activity assays can be combined with other detection assays including surface marker analysis or SP assays when used in practice.

### 3.4 Identifying CSCs via Suspension Culture Assay

Colorectal cancer cells are cultured in a low‐adhesion culture system containing a serum‐free medium supplemented with various cytokines and growth factors that may delay cell differentiation. In this condition, non‐stem colorectal cancer cells fail to sustain and start to anoikis, while CSCs can survive in such a culture system. In this way, cells with stem‐like properties can be selected out [140]. For the in vitro suspension culture system, Sato et al. [141] summarized the requirements for soluble factors of colon adenocarcinoma organoid culture. Basically, the culture medium was made from advanced Dulbecco's modified eagle medium/F12 containing endothelial growth factor and gastrin [141]. Zhou et al. [142] reported the specific conditions that could effectively induce gastrointestinal CSCs, in their experiments, 20 ng/mL EGF + 20 ng/mL bFGF in the culture medium at 30 days of culture obtained the highest fraction of CD44^+^CD133^+^ double‑positive spheroid cells. Compared with other approaches to enrich colorectal CSCs, the suspension culture system has the following advantages [141]. First, the spheroid culture method can efficiently eliminate nonmalignant cell types, such as fibroblasts. This culture system could greatly improve the viability of isolated primary colorectal CSCs without flow cytometry. Furthermore, the suspension culture system can also sustain the characteristics of the original tumor derived from patients, making it possible for personalized drug sensitivity testing and precise treatment [143]. Controversies also arise despite such advantages of the suspension culture system in isolating colorectal CSCs. Long‐time culture makes the culture system susceptible to infection, and the ingredients of the culture medium differ by cancer cell type. Unlike surface markers, researchers can identify CSCs by using specific antibodies and flow cytometry and the suspension culture system still lacks quantification, which may be improved with technological development such as single‐cell sequencing.

### 3.5 Identifying CSCs via Autofluorescence Approach

In 2014, Miranda‐Lorenzo et al. [144] first reported that a small population of cells derived from PDX models of PDAC could be excited with a standard blue laser. Since then, autofluorescence (AF) has been used to identify and isolate CSCs across a wide number of cancers depending on that the CSCs of epithelial tumors contains intracellular vesicles coated with ATP transporters ABCG2, promoting the accumulation of fluorescent vitamin B2 (i.e., riboflavin) within these vesicles, and these AF cells can be identified through fluoresce‐based techniques [144, 145, 146, 147]. Those AF cells exist in adherent cells but were enriched in tumor spheroids. They display stronger invasive potential and enhanced tumorigenic phenotype in vivo compared with nonfluorescent cells. Moreover, those cells also have a higher expression of canonical stem cell markers such as CD133 and CD44. In addition to cancer cells derived from PDAC PDX models, researchers also found that this approach could efficiently identify and isolate colorectal CSCs with strong tumorigenic capabilities [144]. Meanwhile, Alcala et al. [148] also investigated the roles of AF labeled CSCs in resected colorectal tumors and found that the presence of AF CSCs correlated with cumulative incidence of relapse and incidence rate in patients, suggesting the clinical applications of the AF method.

## 4 Signaling Pathways that Regulate the Characteristics and Malignant Behaviors of Gastrointestinal and Hepatic CSCs

Gastrointestinal and hepatic CSCs share some common features with the embryonic stem cells. The canonical pathways involved in cell development and tissue homeostasis are also activated in gastrointestinal and hepatic CSCs, including TGF‐β, Wnt/β‐catenin, JAK/STAT, Notch, and Hedgehog pathways [74, 109]. This section primarily introduces these crucial signaling pathways and their roles in regulating the characteristics and malignant behaviors gastrointestinal and hepatic CSCs.

### 4.1 Wnt/β‐Catenin Signaling

The Wnt/β‐catenin signaling pathway is the canonical Wnt signaling pathway, which exerts the functions of proliferation, differentiation, apoptosis, migration, invasion, and tissue homeostasis [149, 150]. In most cases, Wnt signaling is conserved in mammals, and thus the self‐renewal of cells and homeostasis of tissues can be precisely regulated [151]. While it is reported that the dysregulation of the Wnt/β‐catenin cascade promotes the development and progression of some solid tumors and hematological malignancies [152, 153, 154, 155, 156]. The mutated or hyperactivated Wnt components lead to tumorigenesis. The Wnt/β‐catenin pathway is important for the balance between differentiation and stemness in the stem niches [157], single‐cell transcriptome analysis of colorectal CSCs revealed that Wnt/β‐catenin pathway was among the prominent signaling pathways to maintain stemness [158]. Wnt proteins are usually absent or minimally secreted in normal cells or less aggressive cancer cells. Under this condition, β‐catenin is sequestered in the cytoplasm by a series of destruction complexes (DC) containing Axin, APC, E3 ubiquitin ligase β‐TrCP, and 2 serine‐threonine kinases (CK1a/d and GSK3a/b) [159]. Within the degradation complex, glycogen synthase kinase 3β (GSK3β) and casein kinase 1α (CK1α) mediate the phosphorylation of β‐catenin in the cytoplasm and further promotes its degradation of ubiquitination, leading to the inactivation of downstream Wnt‐target genes [160, 161]. However, in gastrointestinal and hepatic CSCs, Wnt signaling is hyperactivated, and Wnt ligands further bind to its receptors including Frizzled (Fz) protein and phosphorylated lipoprotein receptor‐related protein (LRP). When Wnt ligands are present, their binding to cell surface receptors induces the activation of dishevelled and promotes the aggregation of a complex containing AXIN, GSK3β, CK1, and APC to the receptor location [162]. There, Fz and LRP are phosphorylated and can dock with Axin, leading to the disassembly of DC. Subsequently, the phosphorylation and inhibition of GSK3β ensured an increase in the concentration of β‐catenin in the cytoplasm. Unphosphorylated β‐catenin in the cytoplasm migrates to the nucleus and accumulates, interacting with coactivators such as T‐cell‐specific factor (TCF)/lymphoid enhancement factor binding factor (LEF), Pygopus, and Bcl‐9, triggering Wnt target genes such as c‐Myc, cyclin D1, and CDKN1A. It leads to the upregulation of the TCF/LEF target genes [159, 163]. Although the underlying mechanism of Wnt signaling has been extensively explored, novel insights into the Wnt pathway remain a major scientific problem in gastrointestinal and hepatic CSCs. Various newly discovered activating and inhibitory factors that regulate cell stemness of gastrointestinal and hepatic tumors have been found to exert function through the Wnt pathway.

In gastrointestinal and hepatic cancer cells, several factors could promote Wnt signaling thus exerting functions on stemness. Recently, researchers found that upstream factor LncGata6 could activate Wnt pathway for cell self‐renewal and tumorigenesis. LncGata6 could recruit the NURF complex onto the Ehf promotor to promote the expression of Lgr4/5 and enhance the activation of Wnt, leading to cell self‐renewal and tumorigenesis [164]. Tang et al. [165] found that TM4SF1 could modulate the expression of SOX2 in a Wnt/β‐catenin activation‐dependent manner, thus facilitating the stemness of colorectal cancer cells. Pseudo‐kinase 3 (TRIB3) is increased in some colorectal tumors and associated with poor survival outcomes, Fang et al. [166] found that TRIB3 could interact with β‐catenin and TCF4, recruiting TCF4 and β‐catenin to the promoter region of genes regulated by Wnt. Meanwhile activated β‐catenin increased expression of TRIB3, indicated a positive‐feedback loop and increased the stemness of colorectal cancer cells [166]. In hepatocellular carcinoma, upstream factors can also modulate cancer stemness via activating Wnt signaling pathway. For instance, EPHB2 could activate SRC/AKT/GSK3β/β‐catenin signaling cascade and regulate hepatic cancer stemness and Sorafenib resistance [167]. Additionally, aging is also an activating factor for Wnt. Aging can epigenetically silence multiple inhibiting genes of Wnt, thereby facilitating Wnt signaling for colorectal cancer stemness and tumorigenesis [168]. Besides upstream genes that play the role of activating Wnt, some downstream factors of Wnt may indicate stemness. Stem cells antigen‐1, the downstream target gene of β‐catenin, as a cell surface marker, was enriched for a CSC‐like subpopulation in a primary mouse model of gastric cancer [169]. Interestingly, researchers also found that APC mutant in Wnt signaling may facilitate the clonal advantage of CSCs in colorectal cancer via affecting TME. Mechanistically, APC mutation internally hyperactivates Wnt signaling for self‐growth; it also causes CSCs to secrete high levels of the Wnt antagonist NOTUM. This protein acts as a molecular weapon: it diffuses into the local environment and suppresses WNT signaling in surrounding wild‐type cells. Consequently, these healthy cells are forced to stop proliferating and differentiate, effectively being pushed out of the stem cell niche, which gives Apc‐mutant cells a clonal advantage, allowing them to colonize a crypt. Crucially, inhibiting NOTUM—either genetically or pharmacologically—prevents this mutant expansion and adenoma formation and points a pathway to inhibit CSCs in colorectal cancer [170].

In addition to factors that may stimulate the Wnt pathway activation, potential inhibiting factors are also discovered by researchers. Ordóñez‐Morán et al. [171] found that HOXA5 encodes a class of transcription factors and serves as an antagonist against Wnt to control intestinal cell fate. Mechanistically, HOXA5 directly feeds back onto the Wnt signaling pathway, establishing a system of mutual antagonism in which HOXA5 represses Wnt signaling and Wnt pathway activity prevents HOXA5 expression. In this way, HOXA5 induction can eliminate colorectal CSCs and prevent metastasis [171]. In order to control gastrointestinal and hepatic cancer stemness caused by Wnt, some optimal inhibitors in gastrointestinal and hepatic cancer were also selected out. LGK‐974 is a porcupine (PORCN)‐selective inhibitor that blocks Wnt signaling and tumor growth in vivo [172]. ETC‐159 is also a small molecule PORCN (upstream regulator of Wnt) inhibitor with efficacy in preclinical models of RSPO translocated colorectal cancer [173]. The ongoing clinical trials targeting Wnt signaling in gastrointestinal and hepatic tumors includes vitamin (NCT02603757), curcumin (NCT02724202), aspirin (NCT02607072), and LGK‐974 (NCT01351103) [174].

**FIGURE 2:**
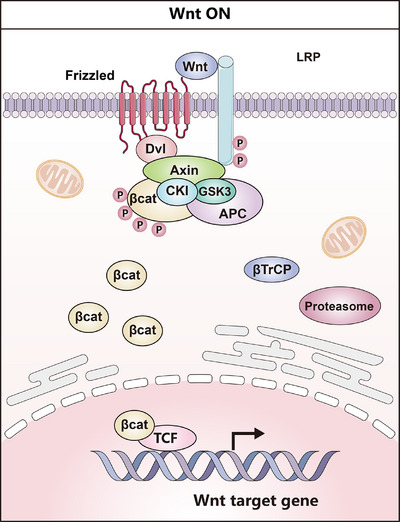
Ideograph for Wnt signaling activation in gastrointestinal CSCs. Binding of Wnt to its receptors induces the association of Axin with phosphorylated lipoprotein receptor‐related protein (LRP). The destruction complex (DC) falls apart, and β‐catenin is stabilized, subsequently binding TCF in the nucleus to upregulate target genes.

In summary, the Wnt/β‐catenin pathway is a crucial, conserved signaling cascade regulating cell proliferation, differentiation, and tissue homeostasis. Dysregulation of this pathway promotes tumorigenesis in solid tumors and hematological malignancies. The exploration of Wnt‐related mechanisms and novel Wnt/β‐catenin regulators deepens the understanding of gastrointestinal and hepatic CSCs to lighten the development of novel drugs for the elimination of CSCs. Ideograph for Wnt signaling activation in gastrointestinal and hepatic CSCs was also shown in Figure 2.

### 4.2 TGF‐β Signaling

The TGF‐β pathway is an emerging mechanism for CSCs. As a member of cytokines, TGF‐β is involved in multiple cellular processes, such as cell proliferation, differentiation, migration, apoptosis, and stemness [175]. The canonical TGF‐β pathway begins with the secretion of the latent complex containing TGF‐β, latent TGF‐β binding protein (LTBP), and latency‐associated peptide (LAP) [176]. After proteolytic activation, the latent complex is destructed, and TGF‐β can be released and bind with the type II TGF‐β serine/threonine kinase receptor. Subsequently, the type I TGF‐β serine/threonine kinase receptor is phosphorylated and mediates the activation of downstream Smad proteins. The hetero‐oligomeric complex with Smad4 protein is formed by activated Smad2 and Smad3 proteins, which can translocate to the nucleus and enhance downstream targeted gene transcription [175, 177]. The TGF‐β pathway has been found to be frequently dysregulated in gastrointestinal tumors [175]. Additionally, the TGF‐β signaling pathway plays a key role in maintaining the CSCs niche. Yusra and Yokozaki [178] reported that *TGFBR1* and *TGFBR3* were highly expressed in CD133^+^ colorectal cancer cells, indicating that colorectal CSCs are characterized by the activation of the TGF‐β pathway. In hepatocellular carcinoma, the expression of TGF‐β is associated with partial EMT augments, mesenchymal genes, CD44 and CD133, and it maintains the activation of epithelial‐related genes [179]. Nakano et al. [117] have found that the enhanced TGF‐β signaling pathway and its downstream target TWIST1 are crucial for the de‐differentiation of colorectal cancer cells and TWIST is highly expressed in undifferentiated CD44^+^ organoids. Similarly, in hepatic cancer cells, TGF‐β may induce EMT to trigger the potential to express stem cell genes for stemness, migration and invasiveness [180, 181]. Alongside the canonical TGF‐β signaling in CSCs, researchers also found some novel mechanisms that exert stemness functions via TGF‐β signaling. Zhang et al. [182] found that MKRN1, an E3 ubiquitin ligase, could lead to the degradation of Smad nuclear‐interacting protein 1, which inhibits TGF‐β signaling, thus promote the stemness and metastasis of colorectal cancer cells. However, it is important to emphasize the dual function of TGF‐β, sometimes it inhibits, and sometimes it promotes the progression of CSCs. Interestingly, in the early stages of cancer, TGF‐β inhibits the tumor, but in the advanced stages, TGF‐β promotes tumor growth and survival [183]. In one way, TGF‐β induces EMT and attenuates the antitumorigenic effects of dendritic cells, NK cells, CD8^+^ T cells, and CD4^+^ T cells, which can be further subdivided into proinflammatory TH1 and anti‐inflammatory TH2 cells. On the other hand, TGF‐β also inhibits ALDH1 on CSCs, thereby limiting their self‐renewal capacity and halting tumor progression. The pro‐ and anti‐inflammatory effects of TGF‐β make TGF‐β a double‐edged sword to CSCs and related solid tumors [184, 185, 186, 187].

**FIGURE 3:**
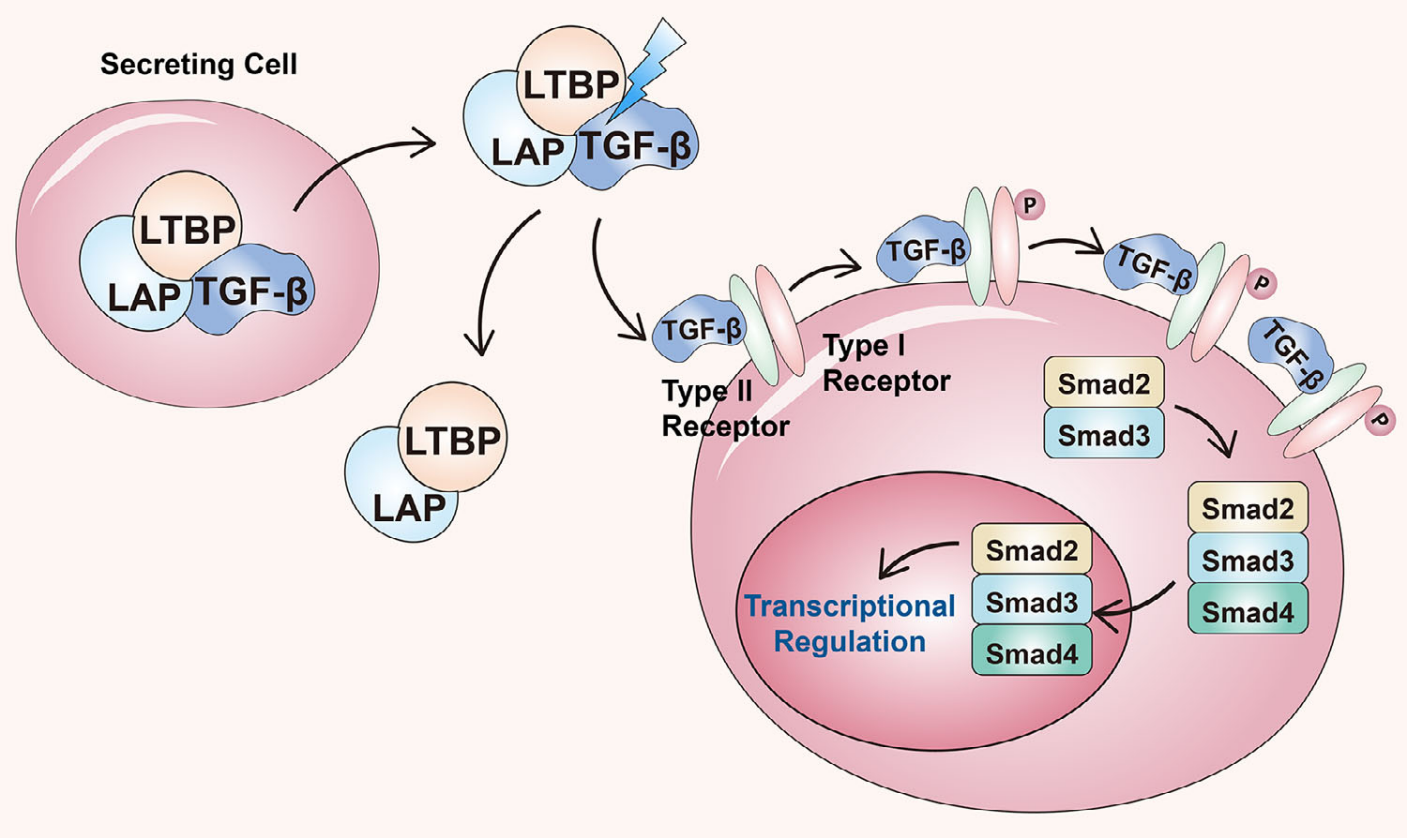
Ideograph for TGF‐β signaling activation in gastrointestinal CSCs. Activation of TGF‐β protein: The latent complex comprised of three proteins, LAP: latency‐associated peptide; LTBP: latent TGF‐β binding protein, TGF‐β: transforming growth factor beta, is secreted into the extracellular matrix. Following proteolytic activation, the TGF‐β cytokine is released from the latent complex and activated. The active TGF‐β protein binds with the type II TGF‐β receptor, which then recruits and phosphorylates the type I TGF‐β receptor. The active complex recruits and phosphorylates the Smad2 and Smad3 proteins that form a hetero‐oligomeric complex with Smad4 protein. The active complex moves into the nucleus where it interacts with transcriptional factors to regulate gene expression.

The TGF‐β signaling pathway also affects the TME. For instance, CAFs can increase the frequency of colorectal CSCs driven by the TGF‐β signaling pathway [188]. Tauriello et al. [189] reported that TGF‐β can promote T cell exclusion and facilitate the immune suppression of colorectal cancer. Currently, clinical trials with TGF‐β inhibitors such as SAR4349459 (NCT03192345) and Bintrafusp alfa (NCT03436563) have shown that targeting TGF‐β signaling is a promising therapeutic strategy for patients with colorectal cancer [190]. In short, the TGF‐β pathway is a key regulator of CSCs in gastrointestinal and hepatic tumors, influencing proliferation, differentiation, migration, and crucially, stemness. The TGF‐β pathway is frequently dysregulated in gastrointestinal and hepatic CSCs and plays a vital role in maintaining CSC self‐renew, Furthermore, it could modulate antitumor immunity response as well as reshape the TME. Due to its CSC promoting effects in advanced cancer, TGF‐β inhibitors are promising therapeutic candidates currently in clinical trials. Ideograph for TGF‐β signaling activation in gastrointestinal CSCs was shown in Figure 3.

### 4.3 JAK/STAT Signaling

**FIGURE 4:**
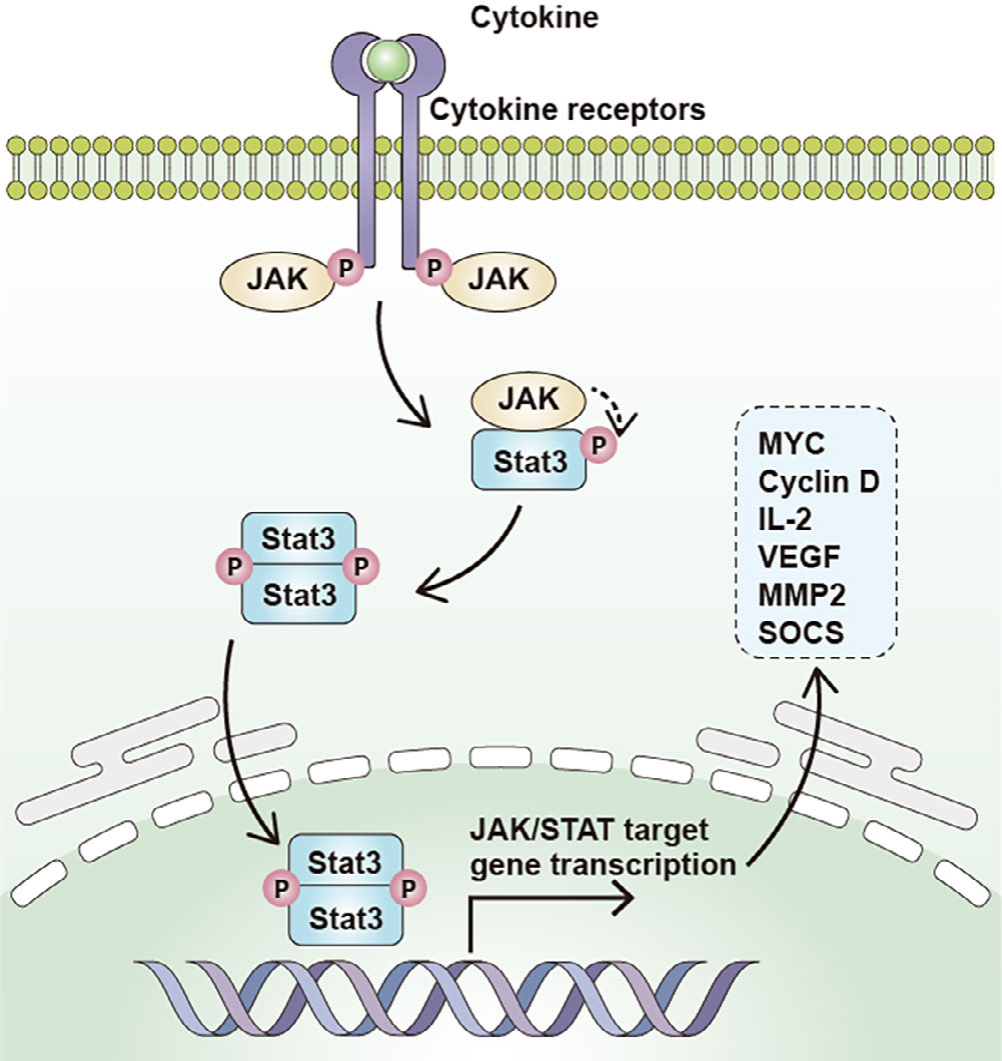
Ideograph for JAK/STAT signaling activation in gastrointestinal CSCs. JAK/STAT signaling starts with the interaction of cytokines or growth factors with their receptors, inducing the dimerization/oligomerization of these receptors and consequent activation. Activated JAKs auto‐phosphorylate and phosphorylate their associated receptors. Therefore, cytoplasmic STATs bind to phosphorylated receptors and undergo homodimerization or heterodimerization after their phosphorylation, and they are able to translocate to the nucleus and activate the transcription of target genes.

The JAK/STAT pathway is an oncogenic signaling pathway in various types of cancer, which is closely related to tumor growth, metastasis, and immune surveillance [191, 192]. JAK/STAT signaling is involved in the maintenance of CSC phenotype in various solid tumors, including breast cancer, esophageal cancer, glioblastoma, and colorectal cancer [191, 193, 194]. The JAK/STAT pathway begins with the release of upstream cytokines, including interferon‐α (IFNα), interferon‐γ (IFNγ), and IL‐6 [195]. Once these cytokines bind with their receptors, the JAK protein is phosphorylated followed by the phosphorylation of STAT3, leading to the translocation of the activated STAT3 dimer to the nucleus. In the nucleus, the STAT3 dimmer binds with the promoter of various oncogenic genes to mediate tumor progression [196]. Abnormal activation of JAK/STAT3 leads to transcriptional upregulation of various oncogenic proteins, including Mcl‐1, Bcl‐2, Bcl‐xl, survivin, Cyclin D1, c‐Myc, MMP2, SOCS. and VEGF, leading to tumor malignant development [197]. The IL‐6/JAK/STAT3 activation also enhances metastasis ability of cancer cells via induction of EMT in gastrointestinal cancers by the upregulation of EMT‐inducing transcription factors such as EMT‐transcriptional factors, such as SNAIL, ZEB1, JUNB, and TWIST‐1 [198]. Chaker et al. [199] revealed that transducing the canonical stemness factors of OCT4, SOX2, KLF4, and c‐Myc in murine hepatocytes could shift hepatocytes into Lgr5^+^ hepatocytes with self‐renew capacity dependent on IL6/JAK/STAT activation. CD44 is a well‐known hallmark for CSCs; except for the hallmark functions, CD44 can also be internalized and translocated into the nucleus to bind with the promoter of multiple targeted genes. In this case, acetylated STAT3 is required as a partner of CD44 protein to bind with the promoter of the target genes, hence maintaining the stemness of CD44^+^ cells [200]. Meanwhile, Park et al. [201] revealed that JAK2 is preferentially highly expressed in CD44v6‐positive colorectal CSCs after radiotherapy, accompanied by the phosphorylation of STAT3 protein. Subsequently, they found CCND2 was directly activated by JAK2/STAT3 axis, which served as a critical aspect to promote the resistance of colorectal cancer to radiotherapy and knock down of CCND2 significantly abolished the self‐renewal activity of colorectal CSC group [201]. In the research of Toh et al. [202], JAK/STAT activation has been identified as a crucial part of inducing SP and CD44^+^ tumorigenic cells in hepatocellular carcinoma; using JAK/STAT inhibitors (TG101209 and AZ960) could effectively induce apoptosis in hepatic CSCs as well as delay tumor formation. Meanwhile, miR‐500a‐3p targets multiple negative regulators of the JAK2/STAT3 signaling pathway, such as SOCS2, SOCS4, and PTPN, in hepatic CSCs, leading to constitutive activation of STAT3 signaling [203]. Based on this context, it is required to develop more promising pharmacological inhibitors targeting JAK/STAT signaling to effectively eliminate gastrointestinal CSC populations thought to be related to tumor metastasis, relapse, and therapeutic resistance. Ideograph for JAK/STAT signaling activation in gastrointestinal and hepatic CSCs was also shown in Figure 4.

### 4.4 Notch Signaling

**FIGURE 5:**
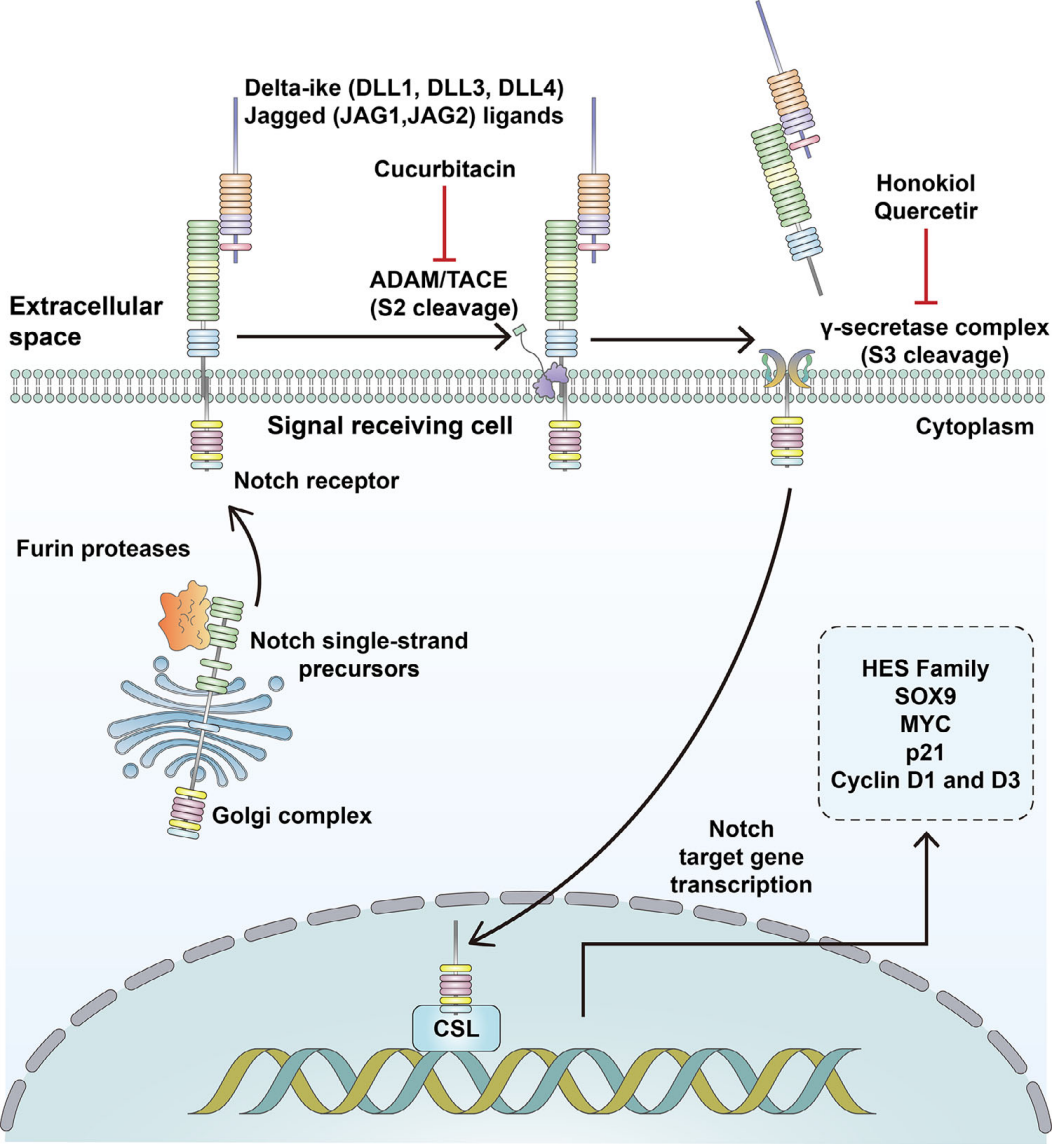
Ideograph for Notch signaling activation in gastrointestinal CSCs. DLL1, DLL3, and DLL4 and Jagged ligands (JAG1 and JAG2) expressed on the cell surface can induce signaling in adjacent cells expressing their cognate receptors Notch1–4. Ligand binding promotes sequential cleavage of the Notch receptors by ADAM/TACE enzymes (S2 cleavage) and then γ‐secretase (S3 cleavage), resulting in release the NICD, which interacts with transcriptional regulators in the nucleus to instigate a Notch gene‐expression profile. Notch target genes, in turn, regulate pivotal cell‐fate choices.

The Notch signaling pathway is a conserved cell‐fate‐determination pathway that is involved in various aspects of cancer progression including stemness, angiogenesis, and antitumor immunity [204, 205]. Similar to TGF‐β signaling pathway, Notch signaling pathway also serves a dual effect to cancer, depending on tissue type or genetic mutation [206]. For example, depending on the TME, Notch signaling pathway is typically downregulated in prostate, skin, lung, liver, and some breast cancers, while Notch signaling pathway is upregulated in gastric, colon, pancreatic, and other breast cancers [207]. The canonical Notch signaling transduction begins with the Delta/Serrate/Lag‐2 (DSL) family ligands binding to Notch receptors. The Notch receptor is subsequently cleaved by a metalloprotease of the ADAM (a disintegrin and metalloprotease) family, then cleaved by γ‐secretase and the Notch intracellular domain (NICD) is translocated to the nucleus to enhance the transcription of *Hairy and Enhancer of split 1* (*HES1*) [208]. The downstream target of Notch signaling pathway includes Myc, cyclin D3, and the HES family of genes [209]. Notch signaling pathway has been identified as a key regulator of gastric and intestinal stem cell function, and Notch signaling activation is associated with enhanced stem‐like properties and chemoresistance [210]. FBXW7, an E3 ubiquitin ligase, modulates Notch expression and mediates Notch degradation. The increased Notch activity can be observed in FBXW7‐mutated patients. In hepatocellular and colorectal carcinomas, FBXW7 under‐regulation affects c‐Myc and NOTCH1 expression levels, which can be directly correlated to cell proliferation, migration, invasion, cancer stemness, and hence poor prognosis [211]. A recent study on gastric cancer also revealed that the low expression of FBXW7 correlates with poor response to chemotherapy, metastatic potential as well as poor prognosis [212]. Delta‐like ligand 4 (DLL4) serves as the upstream ligand of Notch signaling pathway and Segami et al. [213] have found its function in maintaining gastric CSCs characteristics and regulating tumorigenesis. MAGP2, a component of ECM, was found to be associated with the stemness of gastrointestinal cancer via affecting Notch signaling pathway [214]. ZNF217, a member of the zinc finger transcription factor family, dual‐luciferase reporter assay result suggests its roles in targeting Notch1 to activate Notch signaling as well as promoting colorectal cancer stemness [215]. Inducible nitric oxide synthase (iNOS) is associated with the stemness of CD24^+^CD133^+^ hepatic CSCs, researcher found that iNOS could activate TACE/ADAM17 and subsequently trigger Notch signaling to promote stemness of hepatic cancer cells [216]. In the study of Demitrack et al. [217], researchers have found that the aberrant in vivo Notch manipulation affected the efficiency of organoid initiation from glands and single Lgr5^+^ GFP stem cells, also suggesting the regulation of stem cells function. Apart from the regulatory mechanisms of Notch signaling on gastrointestinal and hepatic CSCs, researchers also put their focus on strategies that may inhibit the activation of Notch signaling pathway. RO4929097 is a γ‐secretase inhibitor developed by Roche and is now being tested in phase II clinical trials (NCT01116687); however, as a mono‐therapeutic agent, no significant response was observed in patients with colorectal cancer [218, 219]. Traditional Chinese medicine, Honokiol, may also inhibit activated Notch signaling, the combination of Honokiol and ionizing radiation led to a reduction in the expression of CSC marker and Notch signaling in xenograft tissues, as well as significant suppression of tumor growth [220]. In summary, the Notch signaling pathway plays a complex, context‐dependent role in cancer progression, exhibiting both tumor‐suppressive and oncogenic effects depending on the tissue and genetic mutations. Its canonical activation involves ligand binding (DSL family), sequential cleavage by ADAM metalloproteases and γ‐secretase, releasing the NICD to translocate to the nucleus and activate target genes like HES1, Myc, and cyclin D3. Notch activation is crucial for maintaining CSCs and promoting chemoresistance, particularly in gastrointestinal and hepatic cancers. Potential inhibitors of Notch signaling may provide novel strategies against CSCs. Ideograph for Notch signaling activation in gastrointestinal CSCs was shown in Figure 5.

### 4.5 Hedgehog Signaling

Under the normal condition, the Hedgehog pathway is critical for tissue homeostasis and embryonic development [221]. Hedgehog signaling begins with the binding of Hedgehog ligands containing Desert hedgehog (DHH), Indian hedgehog (IHH), or Sonic hedgehog (SHH) to their Patched (PTCH) transmembrane receptors on Smoothened (SMO) [222, 223]. This binding can relieve the inhibitory effect on SMO and activate the signaling transduction cascade through the nuclear translocation of GLI transcription factors and the expression of Hedgehog target genes, including MYC, CCND1, CCND2, FOXM1, BCL‐2, SNAIL, ZEB, NANOG, SOX2, IL‐6, IL‐1β, and TNF‐α. In gastrointestinal and hepatic CSCs, the Hedgehog pathway also plays a key role in maintaining the stem‐cell phenotype. IHH inhibition leads to impaired self‐renewal capability and increased sensitivity to chemotherapy in gastrointestinal tumors [224]. The tumor suppressor RUNX3 can bind with GLI1 and promote its ubiquitination, thus resulting in an inversely expression of RUNX3 and GLI1. Furthermore, in colorectal cancer tissue, the expression of RUNX3 was disabled, leading to elevated expression of GLI1, activation of Hedgehog signaling and increased ratio of Epcam^+^CD133^+^ CSCs, indicating that the RUNX3‐mediated pathway restricts the oncogenic effect of Hedgehog signaling [225]. The research of He Zhou et al. [226] suggested that novel lncRNA (lncRNA–cCSC1) can modulate the self‐renewal capacity and drug‐resistant capacity of colorectal CSCs via activation of the Hedgehog signaling pathway. Hedgehog signaling pathway also participates in the proliferation and invasiveness of hepatic CSCs. In hepatocellular carcinoma, circIPO11 recruits TOP1 to GLI1 promoter to trigger its transcription, leading to the activation of Hedgehog signaling and providing necessary gene profiles for hepatic CSCs [227]. Chondroitin sulfate synthase 1 (CHSY1), the enzyme that mediates the polymerization step of chondroitin sulfate, is a critical mediator of malignant character in hepatocellular carcinoma, also exerts its function via activating Hedgehog signaling pathway. Inhibiting hedgehog pathway with vismodegib can effectively abrogate the malignant behaviors caused by CHSY1 [228]. Similarly, applying KAAD‐cyclopamine (a specific inhibitor of Hedgehog pathway) could lead to significant attenuation in invasiveness and motility of hepatocellular carcinoma cells [229, 230, 231, 232, 233].

**FIGURE 6:**
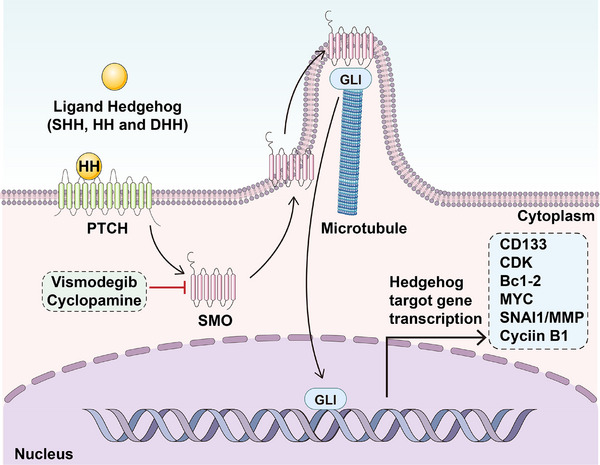
Ideograph for Hedgehog signaling activation in gastrointestinal CSCs. The Hedgehog signaling pathway begins with the binding of Hedgehog ligands containing Desert hedgehog (DHH), Indian hedgehog (IHH), or Sonic hedgehog (SHH) to their Patched (PTCH) transmembrane receptors. Without Hedgehog ligands, on PTCH interacts with SMO and inhibits the activity of SMO; Hedgehog ligands binding to PTCH release its inhibitory effects on SMO, release GLI into cell nucleus, and facilitate the expression of Hedgehog target genes.

Based on the crucial roles of Hedgehog pathway, several inhibitors of Hedgehog pathway were already developed. The first United States Food and Drug Administration (US FDA)‐approved Hedgehog inhibitor for clinical use is vismodegib, which is now applied to metastatic pancreatic cancer as well as mCRC [234, 235]. Cyclopamine, a naturally occurring teratogenic alkaloid, disrupts cholesterol biosynthesis and specifically antagonizes the SHH signaling pathway through direct interaction with SMO. Batsaikhan et al. [236] have found that mRNA level of surface markers and stemness genes decreased significantly after the administration of cyclopamine in HCT116 colorectal tumor spheres. Sonidegib is another Hedgehog pathway inhibitor and is now being tested on patients with advanced hepatocellular carcinoma [237]. In short, the Hedgehog pathway, crucial for development and homeostasis, is activated in gastrointestinal and hepatic CSCs to maintain stemness. Ligands (SHH, IHH, DHH) bind PTCH receptors, releasing inhibition of SMO. This action leads to GLI transcription factor activation and expression of related target genes. Hence finding potential regulatory targets as well as seeking novel inhibiting drugs are promising strategies to overcome gastrointestinal and hepatic CSCs. Ideograph for Hedgehog signaling activation in gastrointestinal CSCs was summarized and presented in Figure 6.

## 5 Therapeutic Strategy via Targeting Gastrointestinal and Hepatic CSCs

The persistence of CSCs presents a major challenge in the treatment of gastrointestinal and hepatic malignancies, as these cells are inherently resistant to conventional therapies and drive tumor relapse. To address this, research has focused on developing targeted strategies that specifically aim to eliminate the CSC population. We begin by discussing the approach of targeting CSC‐specific surface biomarkers (e.g., CD44, EpCAM, CD24) using biologic agents such as monoclonal antibodies (mAbs), antibody–drug conjugates, and CAR‐T cells. Subsequently, we delve into the inhibition of key developmental signaling pathways (e.g., Wnt/β‐catenin, TGF‐β, JAK/STAT, and Hedgehog) that are constitutively activated in CSCs and are essential for their self‐renewal. For each strategy, we evaluate the preclinical evidence and critically appraise the subsequent clinical trial results, highlighting both the promises and the challenges encountered in translating these novel therapies into the clinical practice.

### 5.1 Targeting Surface Biomarkers of Gastrointestinal and Hepatic CSCs

Targeting CSC‐specific surface biomarkers has become a promising strategy for eradicating gastrointestinal CSCs. Strategies targeting CSC‐specific surface biomarkers include mAbs, peptide mimetics, and pharmacological inhibitors, and some of the strategies are being studied in preclinical and clinical trials. Strategies targeting CSC‐specific surface biomarkers conjugated to other antitumor strategies were also being developed for gastrointestinal and hepatic cancer therapy.

In the development of CD44‐targeted antitumor strategy, doxorubicin modified with anti‐CD44mAb exhibited pronounced increase in cellular uptake and elevated intracellular doxorubicin concentration than doxorubicin in CD44‐positive colorectal cancer model [238]. Another phase I trial of RG7356, a recombinant anti‐CD44 IgG1 humanized mAb, was conducted in patients with metastatic CD44‐positive solid tumors, and RG7356 was well tolerated with moderate therapeutic efficacy [239]. CD44 variant domain 6 (CD44v6) is a preciously discovered biomarker for colorectal CSCs, which has been exploited as a promising target for eradicating colorectal CSCs. CD44v6‐targeted polymeric micelles loaded with niclosamide, an anti‐CSC agent, exhibited evident therapeutic efficacy against colorectal CSCs [240]. Screening analysis have found silibinin as a drug targeting CD44v6. Silibinin and 5‐FU could also synergistically impede stemness of colorectal CSCs [241]. Multiple anti‐EpCAM mAbs have been introduced and tested in patients with gastrointestinal tumors. Edrecolomab is an IgG2A mAb against EpCAM, whereas edrecolomab showed limited efficacy in the phase III studies of colorectal cancer. Furthermore, Li et al. [242] developed CAR‐T therapy targeting EpCAM, which was well tolerated and exhibited notable antitumor efficacy (NCT02915445). CD24, a well‐established surface biomarker of CSC, has been exploited as a promising therapeutic target for antitumor therapy. SWA11, a newly developed anti‐CD24 mAb, significantly blocked tumor growth of colorectal cancer [243]. G7, another mAb targeting CD24, exhibited moderate antitumor efficacy via the inhibition of STAT3 signaling pathway by dually blocking CD24 and EGFR [244]. Anti‐CD24 SWA11 mAb has been developed to be conjugated with exotoxin, which significantly induced death of tumor cells without toxicity to normal tissues [245].

### 5.2 Targeting Activated Signaling Pathways of Gastrointestinal and Hepatic CSCs

Multiple signaling pathways participate in the regulation of survival and stemness of CSCs. Currently, the strategies targeting activated signaling pathways involved in gastrointestinal CSCs mainly include Wnt/β‐catenin, TGF‐β, JAK–STAT, and Hedgehog signaling pathways.

The Wnt/β‐catenin signaling pathway is critical for self‐renewal and survival of CSCs. Currently, therapeutics targeting Wnt/β‐catenin pathways include ligand/receptor‐targeted agents, PORCN inhibitors, tankyrase (TNKS) inhibitors, and β‐catenin inhibitors. Ligand‐targeted agents are able to bind with ligands involved in Wnt/β‐catenin pathway, such as anti‐FZD1/2/5/7/8 mAb (vantictumab) and anti‐RSPO3 mAb (rosmantuzumab) [246]. These mAbs have been investigated in preclinical anticolorectal cancer studies. PORCN inhibitors impaired WNT secretion to block WNT signaling pathway, the representative drugs are LGK‐974 and ETC‐159 [172, 173]. TNKS inhibitors could block WNT‐independent signaling cascades. The potent and selective TNKS1/2 inhibitor G007‐LK significantly inhibited Wnt/β‐catenin signaling in models of colorectal cancer, showing therapeutic efficacy in Wnt/β‐catenin pathway mutation‐driven colorectal cancer. However, G007‐LK led to intestinal toxicity resulted from inhibition of Wnt/β‐catenin signaling in intestinal crypts [247]. A novel TNKS inhibitor JW55 directly inhibited the PARP domain of TNKS1/2, leading to increased degradation of β‐catenin to reduce colorectal tumor growth in conditional APC‐mutant mice model [248].

TGF‐β signaling pathway has been found to be hyperactivated in the colorectal CSCs [249]. Preclinical studies proposed that targeting TGF‐β signaling pathway may be a promising strategy for patients with colorectal cancer. And some of therapies have been tested in clinical trials with limited success. Vactosertib (EW‐7197) is a selective TGF‐β receptor I inhibitor, blocks TGF‐β signaling pathway, and shows great antitumor efficacy in multiple tumor types including colorectal cancer. Vactosertib has shown potent antitumor effects against colorectal cancer as a monotherapy or in combination with 5‐fluorouracil in colorectal cancer [250].

JAK/STAT signaling is also important for the maintenance of CSC phenotype in colorectal cancer. Napabucasin is a first‐in‐class tumor stemness inhibitor that targets STAT3. However, napabucasin failed to bring significant difference in overall survival in patients with advanced colorectal cancer (NCT01830621). Notably, napabucasin bring benefit of overall survival in patients with positive p‐STAT3 expression [251]. Another phase I/II trial assessing the efficacy and safety of napabucasin plus pembrolizumab in advanced colorectal cancer. Napabucasin combined with pembrolizumab showed promising antitumor efficacy in patients with advanced colorectal cancer regardless of the status of microsatellite stable [252].

The Hedgehog signaling pathway plays a vital role in the tumorigenesis of colorectal cancer, representing a promising target for antitumor strategy. The seven‐transmembrane protein SMO is critical for the hedgehog signal transduction, thus strategies targeting SMO are promising to block Hedgehog signaling pathway. SMO inhibitor vismodegib was approved by US FDA to treat metastatic basal cell carcinoma [253]. In colorectal cancer, vismodegib has also shown its antitumor activity as a single agent or in combination with other cytotoxic agents in preclinical models of colorectal cancer [253]. In addition, it has been proposed that blocking Hedgehog pathway may lead to increased stromal vascularity and improve penetration of chemotherapeutic agents in preclinical models. However, a phase II study has shown that vismodegib failed to increase the efficacy of standard treatment for advanced colorectal cancer, a total of 199 patients with advanced colorectal cancer were randomized to placebo or vismodegib combined with the standard chemotherapy of advanced colorectal cancer. Notably, no significant benefit was observed in vismodegib combined with standard chemotherapy [235]. In Table 2, we summarized the available preclinical trials on different therapeutic targets [254, 255, 256, 257, 258, 259, 260].

**TABLE 2:** Summary of preclinical trials on different therapeutic targets.

Type	Drug	Mechanism of action	Stage of drug development	References
Small molecule	Vitamin	Wnt/β‐catenin	Not applicable (registration no. NCT02603757; stage III colorectal cancer; completed)	[231]
Small molecule	Curcumin	Wnt/β‐catenin	Early phase I (registration no. NCT02724202; metastatic colon cancer; active NR)	[232]
Small molecule	Aspirin	Wnt/β‐catenin	Phase III (registration no. NCT02607072; nonmetastasized colorectal cancer; recruiting)	[233]
Small molecule	LGK974	Wnt/β‐catenin	Phase I (registration no. NCT01351103; Wnt‐ligands dependent solid tumors; completed)	[234]
Small molecule	SAR4349459	TGF‐β	Phase I (registration no. NCT03192345; malignant solid tumors; completed)	[235]
Small molecule	Bintrafuspalfa	TGF‐β	Phase II (registration no. NCT03436563; colorectal cancer or advanced solid tumors; completed)	[236]
Small molecule	RO4929097	Notch	Phase II (registration no. NCT01116687; solid tumor; completed)	[237]
CAR‐T	EpCAM CAR‐T cells	EpCAM	Phase I (registration no. NCT02915445; advanced solid tumors; active NR)	[219]
Small molecule	Vismodegib	Hedgehog	Phase II (registration no. NCT00636610 Metastatic colorectal cancer; completed)	[212]
Small molecule	Sonidegib	Hedgehog	Phase I (registration no. NCT02151864, advanced or metastatic hepatocellular carcinoma; completed)	[214]
Small molecule	Napabucasin	JAK/STAT	Phase III (registration no. NCT01830621; advanced colorectal cancer; completed)	[228]

## 6 Conclusions and Perspectives

Over the past decades, an enormous proceeding has been made in understanding the characteristics and behaviors of CSCs. The essential roles of CSCs in therapeutic resistance and distant metastasis have been widely recognized. However, how to define the purity of isolated CSCs is still controversial. To date, the flow cytometry method based on stem cell‐specific antigens appears to be the most reliable and widely used method to define CSCs. Concerning stemness testing, xenograft transplantation assay is considered as the golden standard, yet this experiment is relatively difficult to perform and requires in vivo study. Therefore, easier approaches for detecting tumor stemness are required.

Generally speaking, the exploration of gastrointestinal and hepatic CSCs must focus on bridging the gaps between fundamental biological discoveries and clinical applications. One of the most important directions is CSC‐targeted therapy, which may require the application of multiomics analysis, artificial intelligence (AI)‐assisted predictive computational models, PDOs as well as disrupting TME–CSC interactions. Single‐cell techniques have also emerged for the characterization of gastrointestinal CSCs, providing promising opportunities for discovering potential targeted signatures and developing strategies for eradicating CSCs. Recent advances in bioinformatics and machine learning algorithms have enabled researchers to predict CSC vulnerabilities in individual patients. Alongside with the help of AI‐assisted computational model and the popularization of PDOs, the treatment for gastrointestinal and hepatic caner stem cells is stepping into precise medication era. It is worth noting that there is an urgent need for clinical trials evaluating CSC‐targeting agents in combination with traditional treatments. Many CSC‐directed therapies including Notch, Hedgehog, Wnt inhibitors as well as agents affecting TME or metabolic process of CSCs showed potential in preclinical models but failed in clinical practice. This may due to the lack of human tumor heterogeneity in preclinical models compared with original tumor. Additionally, the plasticity of CSCs and their dynamic interaction with niche factors can lead to divergent drug responses that are not accurately predicted in conventional models. Off‐target effect, variabilities between patients, and pharmacokinetic limitations also contribute to the pessimistic clinical results. Hence, to bridge the translational gap, it is crucial to develop more predictive preclinical models, such as humanized mouse models and integrated organoid‐immune cocultures, which can better connect experimental findings to clinical success.

To conclude, exploring the intrinsic properties of CSCs and developing novel methods to eliminate gastrointestinal CSCs may bring great breakthroughs for the current treatment of gastrointestinal and hepatic tumors. Besides, the application of multiomics analysis, AI‐assisted drug screening, PDOs for drug screening, and the development of therapies targeting CSC–TME interactions or plasticity provide more opportunities to develop promising strategies for eradicating gastrointestinal and hepatic CSCs.

## Author Contributions

Jiawen Bu, Mingming Cui, and Yu Zhang: Conceptualization; investigation; software; roles/writing—original draft; writing—review and editing. Yu Lu: Investigation; software; roles/writing—original draft; Xudong Zhu, Fu Peng, and Hong Zhang: Conceptualization; investigation; methodology; software; supervision; validation; roles/writing—original draft; writing—review and editing. All authors have read and approved the final manuscript.

## Funding

This work was supported by National Natural Science Foundation of China (Grant Number: 82403661, 82303557), Open Project Funding of Fujian Provincial Key Laboratory of Innovative Drug Target Research (Grant Number: FJ‐YW‐2023KF01), Open Project Funding of Fujian Key Laboratory of Natural Medicine Pharmacology (Grant Number: FJNMP‐202301), and Doctoral Start‐up Foundation of Liaoning Province (Grant Number: 2023‐BS‐048).

## Ethics Statement

The authors have nothing to report.

## Conflicts of Interest

The authors declare no conflicts of interest.

## Data Availability

The authors have nothing to report.
